# Essentiality of the *Escherichia coli* YgfZ Protein for the In Vivo Thiomethylation of Ribosomal Protein S12 by the RimO Enzyme

**DOI:** 10.3390/ijms24054728

**Published:** 2023-03-01

**Authors:** Torben Lund, Maria Yohanna Kulkova, Rosa Jersie-Christensen, Tove Atlung

**Affiliations:** Department of Science and Environment, Roskilde University, Universitetsvej 1, 4000 Roskilde, Denmark

**Keywords:** S12 D88, 4Fe-4S cluster, Radical SAM, bottom-up proteomics, targeted LC-MS^2^

## Abstract

Enzymes carrying Iron-Sulfur (Fe-S) clusters perform many important cellular functions and their biogenesis require complex protein machinery. In mitochondria, the IBA57 protein is essential and promotes assembly of [4Fe-4S] clusters and their insertion into acceptor proteins. YgfZ is the bacterial homologue of IBA57 but its precise role in Fe-S cluster metabolism is uncharacterized. YgfZ is needed for activity of the radical S-adenosyl methionine [4Fe-4S] cluster enzyme MiaB which thiomethylates some tRNAs. The growth of cells lacking YgfZ is compromised especially at low temperature. The RimO enzyme is homologous to MiaB and thiomethylates a conserved aspartic acid in ribosomal protein S12. To quantitate thiomethylation by RimO, we developed a bottom-up LC-MS^2^ analysis of total cell extracts. We show here that the in vivo activity of RimO is very low in the absence of YgfZ and independent of growth temperature. We discuss these results in relation to the hypotheses relating to the role of the auxiliary 4Fe-4S cluster in the Radical SAM enzymes that make Carbon-Sulfur bonds.

## 1. Introduction

Iron-Sulfur (Fe-S) clusters are present in all three domains of life as cofactors in proteins with important roles in many fundamental processes. The Fe-S clusters participate in electron-transfer reactions, redox reactions, respiration, photosynthesis, intermediary metabolism, DNA repair, gene regulation, and other processes [1,2]. The model organism *Escherichia coli* (*E. coli*) K12 MG1655 encodes, according to EcoCyc [3], 125 proteins containing [4Fe-4S] clusters and 35 proteins with [2Fe-2S] clusters.

The biogenesis of Fe-S proteins is a complex process that is very similar in all organisms; the proteins involved are mostly homologous in bacterial and eukaryotic systems (Py and Barras 2010, [2,4]). In all organisms, the process starts with extraction of the Sulfur atom from cysteine by a cysteine desulfurase, and the Sulfur is then transferred to a scaffold protein where a [2Fe-2S] cluster is formed. For building [4Fe-4S] clusters the [2Fe-2S] is transferred from the scaffold protein to a carrier protein with the aid of a chaperone pair and mediated by ATP hydrolysis [2]. *E. coli* has two Fe-S protein-building systems, Isc and Suf, where the Isc system is considered the main system and Suf an auxiliary system primarily for “emergency” situations like oxidative stress [4]. The bacterial Isc system is homologous to the mitochondrial ISC system and the late part of the assembly of [4Fe-4S] proteins has mainly been studied in humans and yeast. The current picture is that the [4Fe-4S] cluster is generated from two [2Fe-2S] clusters on the ISCA carrier protein with the help of the IBA57 protein [5,6,7] and then inserted either directly or via trafficking proteins into the final acceptor proteins, e.g., aconitase, LipA, etc [2]. IBA57 is like the other components of the ISC system essential in mammals [8]. YgfZ is the bacterial homologue of IBA57 [9,10]. YgfZ is not essential in *E. coli*, but disruption of the *ygfZ* gene leads to cold sensitivity of growth, reduced activity of several [4Fe-4S] enzymes, as well as sensitivity to the oxidative stress agent plumbagin [10,11,12]. The growth phenotypes of the *E. coli ygfZ* mutant are rescued by the IBA57 gene from different eukaryotic organisms including mice [10], indicating that the IBA57 and YgfZ proteins carry out very similar functions in the biogenesis of Fe-S proteins.

**Figure 1:**
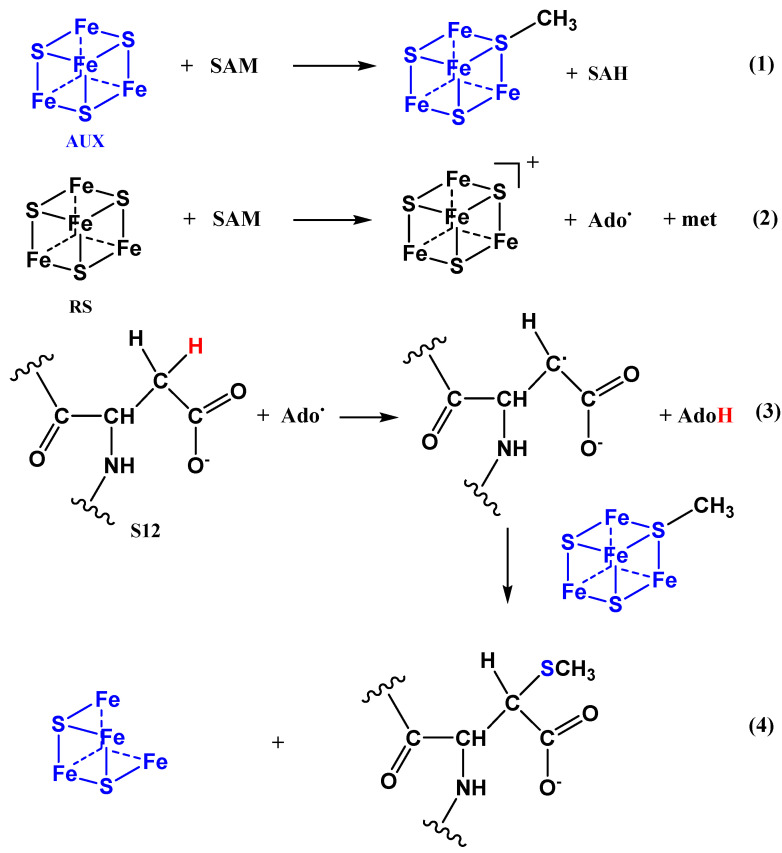
Mechanism of the thiomethylation of the ribosomal S12 protein by the RimO enzyme.

The Radical SAM (S-adenosyl methionine) superfamily of enzymes all contain a [4Fe-4S] cluster (RS) which, in its reduced state, fragments SAM to methionine (met) and a 5′deoxyadenosyl radical (Ado^.^) that removes a hydrogen atom creating a substrate-based carbon radical [13]. *E. coli* has four enzymes that belong to a subclass of RS enzymes that all contain an auxiliary Fe-S cluster (AUX) and carry out a sulfur-insertion reaction [14]. Biotin synthase (BioB) contains a [2Fe-2S] cluster, whereas a [4Fe-4S] auxiliary cluster is present in the other three—lipoyl synthase (LipA), MiaB (a tRNA modifying enzyme), and RimO that thio-methylates ribosomal protein S12 [15,16,17]. MiaB and RimO enzymes both use a second SAM molecule to donate the methyl group [18,19], but the source of the sulfur has been a subject of debate until recently. It has been proposed that the second SAM molecule methylates the “free S” (sulfide ion) in the auxiliary [4Fe-4S] cluster and that this thio-methyl group is then transferred to the radical-activated Carbon in the tRNA/protein substrate [20] (see Figure 1). A recent study of the structures of intermediates in the enzymatic reaction of a MiaB enzyme strongly supports this hypothesis [21]. The MiaB and RimO enzymes perform very similar reactions and are highly homologous [15]. The degree of tRNA thiomethylation is low in the *ygfZ* mutant [10,11,12] suggesting that the sustained activity of the MiaB enzyme requires the presence of the YgfZ protein for insertion, and possibly maintenance, of the [4Fe-4S] clusters.

In this study, we describe the first investigation of the role of the YgfZ protein for the *E. coli* RimO thiomethylase activity, and, in addition, determine the extent of the S12 D88 thiomethylation at different growth phases and growth temperatures. The thiomethylation was determined by a bottom-up proteomic procedure in which the bacterial proteome was digested by trypsin followed by a target LC-MS^2^ analysis of the peptides containing the D88 amino acid.

## 2. Results

### 2.1. Growth Characteristics of the Bacterial Strains

We carried out experiments with the wild type and knockout mutants of *rimO* and *ygfZ* (see the bacterial strain details in Materials and Methods). The bacteria were grown in an LB medium for all experiments. Generation times for the different strains are shown in Table 1. The generation time for the *rimO* mutant was the same as that for the wild type and the growth curves and growth yields were very similar for these two strains (Figure S1).

**Figure 2:**
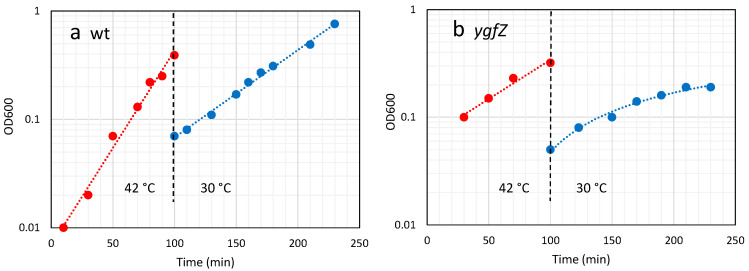
Growth curves for 42 → 30 °C experiments. The cultures were diluted and shifted at the time 100 min. Symbols: red 42 °C; blue 30 °C. (**a**) wt strain; (**b**) *ygfZ* mutant.

The *ygfZ* mutant is cold-sensitive and unable to form colonies at 30 °C [11,12]. Based on this cold-sensitive phenotype we expected that the *ygfZ* mutation could have a different effect on thiomethylation at high versus low temperatures. We therefore did the experiments as temperature shifts, with growth and sampling for LC-MS^2^ analysis at 42 °C, before shifting the bacterial cultures to 30 °C and at the same time diluting to keep the bacteria in a log-growth phase (Figure 2). After two mass doublings at 30 °C, samples were taken for analysis of thiomethylation. The growth of the *ygfZ* mutant was also strongly compromised at 42 °C (Figure 2 and Table 1) and after the shift to 30 °C exponential growth only continued for approximately 2 generations and the cells almost stopped growing 4 h after the temperature shift. The *ygfZ* mutant is prone to picking up suppressor mutations at high temperatures ([11], own unpublished observations). We performed six biological replicates of the *ygfZ* mutant, using two independent isolates of the mutation in our wild type (wt) strain. All six biological replicates had the same growth characteristics indicating that the bacteria had not acquired any suppressor mutations.

### 2.2. MS^2^ Analysis of Thiomethylation of S12

**Figure 3:**
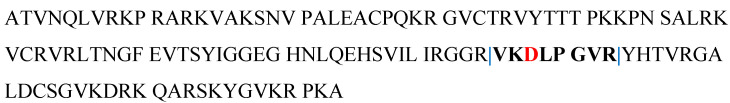
Primary amino sequence of the S12 protein with the thiomethylated aspartic acid **D**88 indicated in red. The two relevant trypsin enzyme cleavages are indicated by dashes and the resulting peptide is in bold face.

The primary amino acid sequence of the *E. coli* S12 protein is shown in Figure 3. The number 88 amino acid in the sequence is aspartic acid (D88) which may be thiomethylated at the β carbon atom as shown in Figure 1. Throughout this article, the thiomethylated aspartic acid is indicated in red (D) and a non-thiomethylated aspartic acid in black (D).

The degree of S12 thiomethylation was obtained by targeted LC-MS analysis of the peptides containing the D88 amino acid. Digestion of S12 with the protease trypsin was followed by a targeted LC-MS^2^ analysis of the peptides containing the D88 amino acid. Treatment of S12 with trypsin is expected to produce the small peptide DLPGVR according to the general trypsin cleavage rules. However, Strader et al. [22] did not find this small peptide and only detected the one miscleaved peptide VKDLPGVR. We have therefore focused our analysis on the detection of VKDLPGVR and its thiomethylated analog VK**D**LPGVR. Initial proteomic experiments indicated the presence of the two miscleaved peptides VKDLPGVRYHTVR and GGRVKDLPGVR. However, we did not find these peptides in the bacterial samples obtained by targeted LC-MS^2^ analysis. HPLC and ESI-MS spectra of the reference peptides corresponding to these two miscleaved, non-thiomethylated peptides are shown in Supplementary Figures S2 and S3, respectively.

**Figure 4:**
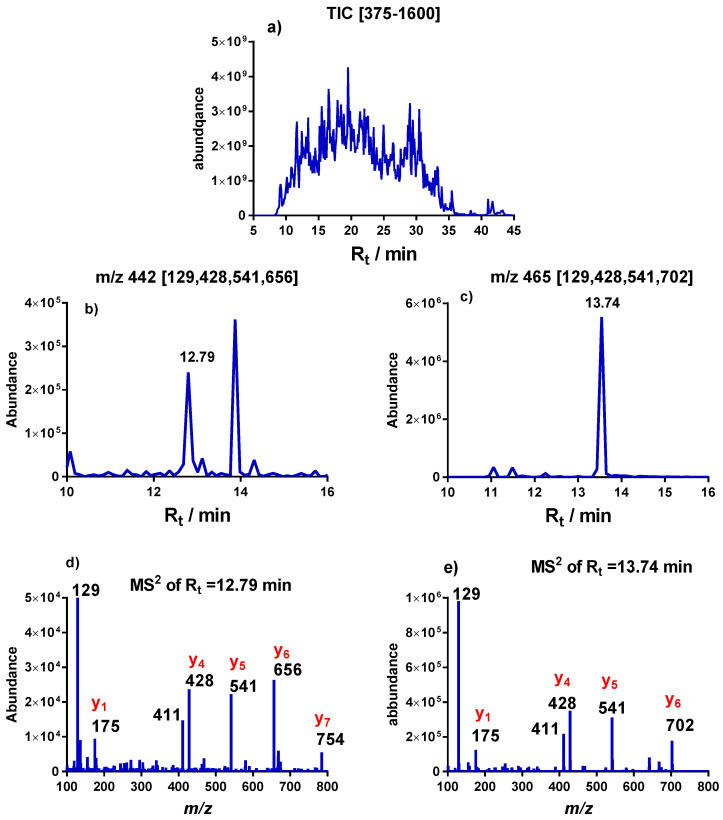
Targeted LC-MS analysis of a trypsin-treated wild type bacterial *E. coli* sample. (**a**) Total Ion Chromatogram (TIC) in the *m/z* interval [375–1600]. (**b**,**c**) Chromatograms obtained with the MS^2^ filters, MS^2^ 442.27@hcd 35 [129, 428, 541, 656] and MS^2^ 465.26@hcd 35 [129, 428, 541, 702], respectively. (**d**,**e**) MS^2^ spectra of the double-charged trypsin peptides of VK**D**LPGVR (R_t_ = 12.79 min) and the thiomethylated VK**D**LPGVR peptide (R_t_ = 13.74 min).

Figure 4 shows a LC-MS analysis of the two target peptides VK**D**LPGVR and VK**D**LPGVR in the trypsin-digested wild type bacterial *E. coli* sample. Figure 4a shows the complex LC-MS^1^ chromatogram of a mixture of the large number of double-charged trypsin peptides of the complete bacterial proteome digest. The two target peptides were extracted from the complex chromatogram by application of two MS^2^ filters with the *m/z* values of the double-charged [M+2H]^+^ ions of the VK**D**LPGVR (*m/z* = 442.27) and VK**D**LPGVR (*m/z* = 465.31) peptides. The MS^2^ filters may be even more selectively designed by only recording the intensities of the MS^2^ fragmentation ions of the two target peptides (see Table 2). Figure 4b,c shows the result of the mass spectrometry filtration process. The peak at R_t_ = 13.74 min in Figure 4c was identified as the VK**D**LPGVR peptide based on its MS^2^ spectrum shown in Figure 4e, which match the MS^2^ spectrum published by [22]. The chromatogram in Figure 4b shows several peaks; however, only the peak eluting at R_t_ = 12.79 min has a MS spectrum identical to the reference peptide VKDLPGVR. Supplementary Figure S4 illustrates the LC-MS^2^ filtration process in more detail and includes additional chromatograms. Table 3 contains retention times and the *m/z* values of the two target peptides.

Table 3 shows the results of the S12 thiomethylation analysis of the wild type (wt), *rimO* and *ygfZ E coli* bacterial strains as a function of growth phases (identified by OD_600_ values of the bacterial cultures), and the growth temperature. For the wild type bacteria, the S12 protein is predominately thiomethylated with a degree of thiomethylation between 94–99% and with small variations between growth phases and temperatures. The *rimO* bacterial mutant lacks the thiomethylation enzyme RimO and the *rimO* bacterial strain is, therefore, not expected to be able to thiomethylate S12. This expectation is clearly supported by our results showing a thiomethylation degree of zero % for the *rimO* mutant in accordance with previous reports [15,22]. Interestingly, the thiomethylation degree of the *ygfZ* bacterial strain is very low, but not zero as for the *rimO* mutant. The *ygfZ* strain has a thiomethylation degree of 0–3% with an average degree of the 12 biological replicates of 2.5%. As observed in the extended result table (Supplementary Table S1), the sum of the chromatographic peak areas of the VK**D**LPGVR and VK**D**LPGVR peaks are reasonably constant.

## 3. Discussion

We have demonstrated here that the ratio between the thiomethylated and non-thiomethylated ribosomal protein S12 can be quantified reliably in whole-cell extracts of *E. coli*. The degree of thiomethylation of S12 was obtained simply using total bacterial cell extracts for trypsination followed by LC-MS^2^ analysis. Previous studies of in vivo S12 thiomethylation used either proteins from purified 30S ribosomal subunit [15,23] or Spa-tagged S12 protein isolated by pull down [22]. Control experiments using pure peptides and the *rimO* mutant showed that our method is very reliable; i.e., we detected no peptide corresponding to the thiomethylated VK**D**LPGVR in the *rimO* mutant, and no peptide corresponding to the expected DLPGVR from complete-trypsin digestion of S12, as observed previously by [22]. Furthermore, we found no other mis-cleaved S12 peptides carrying the DLPGVR sequence in the wt, *rimO* or *ygfZ* mutants besides the one mis-cleaved peptide VKDLPGVR.

The analysis of wt cells showed that S12 was almost fully thiomethylated as found in a study using Spa-tagged S12 [22], and this result was observed independently of growth phase, although there may have been slightly more unmethylated S12 during the true log phase than in the stationary phase (Table 3). This shows that the RimO enzyme activity is not limiting for thiomethylation, even in the fast-growing cells in log phase in rich medium. The slightly higher degree of thiomethylation in the stationary phase is to be expected as the rate of ribosome synthesis becomes much lower as cells approach the stationary phase and the ratio between the RimO enzyme and newly made S12 increases. We also measured thiomethylation at different growth temperatures and found no significant differences in the temperature interval from 30 to 42 °C. All previous analyses used cells grown at the optimal temperature for *E. coli* of 37 °C.

The main aim of our experiments was to investigate the role of the YgfZ protein in activity of the RimO enzyme since the homologous MiaB tRNA thiomethylating enzyme is dependent on YgfZ for normal activity. The *ygfZ* mutant is cold-sensitive [11,12]; we therefore performed the measurements both at permissive and non-permissive temperatures (42 °C and 30 °C), but found that the mutant has almost zero S12-thiomethylation at both growth temperatures. The cold sensitivity may be caused by the presence, at high temperatures, of another protein partially substituting for YgfZ. The result indicates that for the RimO activity, YgfZ is the only protein that can perform the needed function. The effect of the *ygfZ* mutation on RimO activity is, therefore, more pronounced than the effect on MiaB activity, where there is some residual thiomethylation of tRNA (see Table 4) [10,24].

There is a pronounced difference in the requirement of different [4Fe-4S] enzymes for YgfZ. The activity of Tricarboxyl Acid cycle enzymes is only moderately reduced in the mutant [10], whereas there is a strong effect on the two thiomethylating enzymes RimO and MiaB. This could be because of a difference in dependence on YgfZ for insertion of the [4Fe-4S] clusters into the acceptor proteins. In yeast, however, Iba57 was found in vivo to facilitate the transfer of [4Fe-4S] clusters from Isa1 and 2 carrier proteins into virtually all mitochondrial acceptor proteins [5,25]. We, therefore, favor an alternative explanation for the strong effect of *ygfZ* knockout on the activity of RimO (and MiaB), i.e., that it is due to the special feature of the reaction carried out by the Radical SAM enzymes RimO, MiaB, and LipA carrying two [4Fe-4S] clusters. Using in vitro assays containing just the enzyme, a substrate, and SAM, the maximum turnover is one product per enzyme molecule [14,17] because of the use of the auxiliary [4Fe-4S] cluster to provide the S for the thiomethylation/thiolation of the substrate; i.e., the enzyme acts as a substrate in the reaction. Recent studies of the *E. coli* LipA enzyme [26,27] convincingly show that this enzyme acts catalytically when it can be regenerated by inclusion of a loaded [4Fe-4S] carrier protein (NfuA or IscU) in the assay. These studies used isotope labelling to show transfer of the [4Fe-4S] cluster from the carrier to LipA and showed that the labelled Sulfur is subsequently incorporated into lipoic acid. Sustained activity of the MiaB and RimO enzymes requires the repair/replacement of the auxiliary [4Fe-4S] cluster and this may have different requirements than the insertion of the cluster into the de novo synthesized apoproteins; i.e., the requirement for different carrier proteins that are dependent on YgfZ for activity. Unfortunately, there are no studies investigating the effect of the inclusion of a Fe-S carrier protein in the in vitro assays of RimO or MiaB.

The apparent essentiality of YgfZ at low temperatures cannot, however, be explained by the effect on the RimO and MiaB enzymes, since both of these are dispensable for growth and single mutants lacking these enzymes show no growth defects under normal growth conditions [15,28]. Neither can it be due to a lack of lipoic acid, which is essential in all organisms [29], since the cold-sensitivity is also displayed in the LB medium which contains lipoic acid that can be taken up and used by *E. coli*. The cold-sensitivity of growth must be due to effects on other [4Fe-4S] enzymes, probably a combined effect on many different enzymes. It should prove interesting to analyze the proteome of the *ygfZ* mutant grown at different temperatures to perhaps pinpoint the most important [4Fe-4S] cluster proteins affected by the lack of this accessory protein in Fe-S cluster biogenesis.

## 4. Materials and Methods

### 4.1. Materials

Three reference peptides were purchased from the company TAG Copenhagen A/S: VKDLPGVR, VKDLPGVRYHTVR and GGRVKDLPGVR.

### 4.2. Bacterial Strains and Growth Conditions

The bacterial strains used in this study are listed in Table 5. Strains TC5541 and 42 were constructed by phage P1(vir) transduction according to standard genetic procedures [30] selecting for Kan^R^ on LB [30] plates supplemented with 50 μg/mL kanamycin. Wild type and *rimO* strains were grown at 37 °C, while strains carrying the *ygfZ* mutation were grown at 42 °C except when stated explicitly.

For protein extraction and MS analysis the bacteria were grown in LB medium with three or six biological replicates. Three or six overnight cultures in an LB medium were inoculated from single colonies on LB agar plates. Precultures were inoculated from these overnight cultures and grown in a shaking-water bath at the appropriate temperature for at least 4 generations until the experiment was started. For the growth phase experiments, the wild type and *rimO* mutant samples were harvested at OD_600_ approx. 0.25, 0.75 and 2.5. An amount of cells corresponding to 1.5 mg of dry weight was harvested by centrifugation for 4 min at 10.000× *g* at 4 °C. The cell pellets were washed twice with ice-cold 10 mM MgCl_2_ and stored at −20 °C. For the experiments including the *ygfZ* mutant the overnight cultures and pre-cultures were grown at 42 °C. At OD_600_ approx. 0.3 of the 42 °C samples were harvested and the cultures were diluted five-fold into a fresh LB medium and transferred to 30 °C; growth was followed until OD600 again reached 0.3 at which point the 30 °C samples were harvested.

### 4.3. Extraction of Bacterial Protein

The lysis buffer (6 M Guanidine hydrochloride; 100 mM Tris–HCl pH 8.5; 10 mM chloroacetamide (CAA); 5 mM tris(2-carboxyethyl)phosphine (TCEP)) was used to resuspend the cell pellets. The lysates were incubated in the heat block for 10 min at 99 °C after which 1 min sonication followed in order to sheer DNA. The sample’s protein concentration was measured with a nanodrop at 280 nm with a targeted mass of 200 μg of total proteins in each sample. Then, the protein samples were diluted 10 times with 25mM Tris at pH 8 and digested with trypsin (4 μg) using a 50:1 protein:enzyme ratio. Digestion was performed at 37 °C overnight with shaking. The next day, the digestion mixture was quenched with 10% TFA to give a final concentration of ~1% TFA and frozen at −20 °C. Thereafter, samples were centrifuged for 10 min at 4500 to remove non-digested proteins and cell debris. Purification was performed on Waters Sep-Pak^®^ cartridges; Acetonitrile with 0.1% TFA was used for washing and the cartridges were eluted in 40% and 60% ACN, respectively. The SpeedVac vacuum concentrator was used to concentrate samples at 45 °C after which the lysates were resuspended in a 50 µL MS buffer (5% ACN in 0.1% TFA) and protein concentration was measured (Nanodrop, Thermo Scientific, Wilmington, DE, USA) and set to its final value of ~0.25 µg/µL. The samples then underwent LC-MS analysis.

### 4.4. LC-MS Analysis

The LC-MS equipment comprised a Dionex Ultimate 3000 nano-HPLC connected to a LTQ Velos Orbitrap (Thermo Scientific, San Jose, CA, USA). The chromatographic separation was performed with a nano-C18 fused silica column (i.d. = 75 μm, 75 cm long), pulled, and packed in-house with 1.9 μm C18 beads (Reprosil-AQ Pur, Dr. Maisch, Ammerbuch, Germany). The elution of the tryptic peptides was performed with a flow of 250 nL/min and mobile phase A (0.1% formic acid in H_2_O) and B (90% acetonitrile with 0.1% formic acid in H_2_O). The gradient program was as follows: 5% B increased linearly to 25% B in 20 min, from 25 to 50% B in 10 min, from 50 to 95% B in 5 min followed by a 2 min wash and a re-equilibration step. The LTQ Velos Orbitrap was operated in targeted MS^2^ mode with 13 scans performed in each cycle, starting with a full MS scan in positive mode [375.00–1600.00] followed by 12 MS^2^ scans of the ions of the three target peptides and their thiomethylated versions: VKDLPGVR (2^+^, 442.27, 465.26), VKDLPGVRYHTVR (4^+^ 385.71, 397.23; 3^+^ 513.95, 529.31; 2^+^ 770.43, 793.47), and GGRVKDLPGVR (3^+^ 385.22, 400.58; 2^+^ 577.33, 600.37). Full scan mass spectra were recorded in the orbitrap at a resolution of 60,000 at *m/z* 200 with a target value of 1 × 10^6^ and a maximum injection time of 500 ms. HCD-generated fragment ions were recorded in the orbitrap with a resolution at 30,000 over the *m/z* range 50–1600. The maximum ion injection time was set to 100 ms and a target value was set to 1 × 10^4^. Spray voltage was set to +2.2 kV, S-lens RF level at 50, and heated capillary at 300 °C. Normalized collision energy was set at 35 and the isolation window was 1 *m/z.* The data analysis was performed using a combination of the Thermo programs “Free Style” and “Xcalibur”.

## Figures and Tables

**Table 1:** Generation times of the bacterial strains at different growth temperatures.

	wt	*rimO*	*ygfZ*
30 °C	36.1 ± 1.1 min	NT ^a^	59 ± 6.1 min ^b^
37 °C	21.5 ± 0.5 min	22.2 ± 1.0 min	NT
42 °C	18.3 ± 0.2 min	NT	40 ± 1.8 min

^a^ NT: Not tested. ^b^ The bacteria were only growing exponentially for the first two doublings after the shift from 42 °C to 30 °C.

**Table 2:** Mass spectrometry data of the S12 trypsin peptide containing the D88 ^(a)^.

	VKDLPGVR	VKDLPGVR
R_t_/min	12.79	13.74
M_w_	882.5297	928.6117
[M+2H]^+^	442.2727	465.3136
Immonium ion of R	129.1022	129.1022
[R+H]^+^ (y1)	175.1190	175.1189
[PGVR+H]^+^ (y4)	428.2615	428.2614
[LPGVR+H]^+^ (y5)	541.3453	541.3455
[DLPGVR+H]^+^ (y6)	656.3723	702.3597

^(a)^ **D** = D88 is thiomethylated, **D** = D88 is non-thiomethylated.

**Table 3:** Percent S12 thiomethylation in wild type (wt), *rimO*, and ygfZ bacterial strains grown at 30–42 °C and harvested at different bacterial-growth phases ^(a,b,c)^.

Bacterial Strain	Geno- Type	OD_600_	t/°C	Biological Replicates	$\frac{VKDLPGVR}{\mathrm{VKDLPGVR}}$	% S12 Thiomethylation
TC5540	wt	0.2	37	3	20 ± 6	95 ± 2
		0.7	37	3	31 ± 15	96 ± 2
		2.5	37	3	83 ± 17	99 ± 1
TC5542	*rimO*	0.2; 0.7; 2.5	37	3	0	0
TC5540	wt	0.2	30	3	18 ± 5	94 ± 2
		0.2	42	3	40 ± 20	96 ± 3
TC5541	*ygfZ*	0.2	30	6	0.02 ± 0.03	1.5 ± 3
		0.2	42	6	0.03 ± 0.04	3 ± 4

^(a)^ Exponential (OD_600_ = 0.2), late log (OD_600_ = 0.7), and stationary (OD_600_ = 2.5) bacterial-growth phases defined by the optical density of the bacterial cultures at 600 nm. ^(b)^ **D** = the thiomethylated D88 amino acid in S12. ^(c)^ The data in Table 3 are based on an extended table (Supplementary Table S1) showing individual results for the various biological replicates.

**Table 4:** Effect of YgfZ on the RimO and MiaB thiomethylation of S12 and tRNA, respectively ^(a)^.

*E. coli* Strain	$\frac{VKDLPGVR}{\mathrm{VKDLPGVR}}$	$\frac{tRNA-I^{6}A-S-CH_{3}}{\mathrm{tRNA}-I^{6}A}$
Wild type	40	30
*rimO* or *miaB* (knock-out)	0	0
*ygfZ* (knock out) *rimO*^+^ *miaB*+	0.03	2

^(a)^ tRNA data from [10].

**Table 5:** *E.coli* strains.

Strain	Genotype	Reference/Source
MG1655	F^−^ λ^−^ *rph*-1	[31]
TC5540	F^−^ λ^−^ *rph*-1 *tonA*	Spontaneous T1^R^ mutant of MG1655 obtained from Løbner-Olesen
JW2866	Δ*lacZ4787*::(*rrnB-3*) *hsdR514* Δ*araBAD567* Δ*rhaBAD568 ygfZ*::*aphA*	The Keio collection NBRP (NIG, Japan)
JD21892	F^−^ *lacI*^q^ *lacZ*ΔM15 *galK2 galT22* lambda- in (*rrnD-rrnE*)1 *rimO*::(mini-Tn10 Kan^R^)	Transposon insertion disruptant collection NBRP (NIG, Japan)
TC5541	F^−^ λ^−^ *rph*-1 *tonA ygfZ*::*aphA*	This work
TC5542	F^−^ λ^−^ *rph*-1 *tonA rimO*::(mini-Tn10 Kan^R^)	This work

## Data Availability

Data is contained within the article or Supplementary Material.

## Supplementary Materials

The following supporting information can be downloaded at: https://www.mdpi.com/article/10.3390/ijms24054728/s1.
