# Supplementary material for: Essentiality of the Escherichia coli YgfZ Protein for the In Vivo Thiomethylation of Ribosomal Protein S12 by the RimO Enzyme
Source: Int J Mol Sci. 2023 Mar 1;24(5):4728. doi: 10.3390/ijms24054728 (PMC10002905; doi:10.3390/ijms24054728)
Supplement: Supplementary file 1 [file ijms-24-04728-s001.zip › ijms-2187963-supplementary.pdf]

Supplementary

Essentiality of the *Escherichia coli* YgfZ protein for the in vivo thio-methylation of ribosomal protein S12 by the RimO enzyme.

Torben Lund, Maria Yohanna Kulkova, Rosa Jersie-Christensen, and Tove Atlung*

Department of Science and Environment, Roskilde University, Denmark

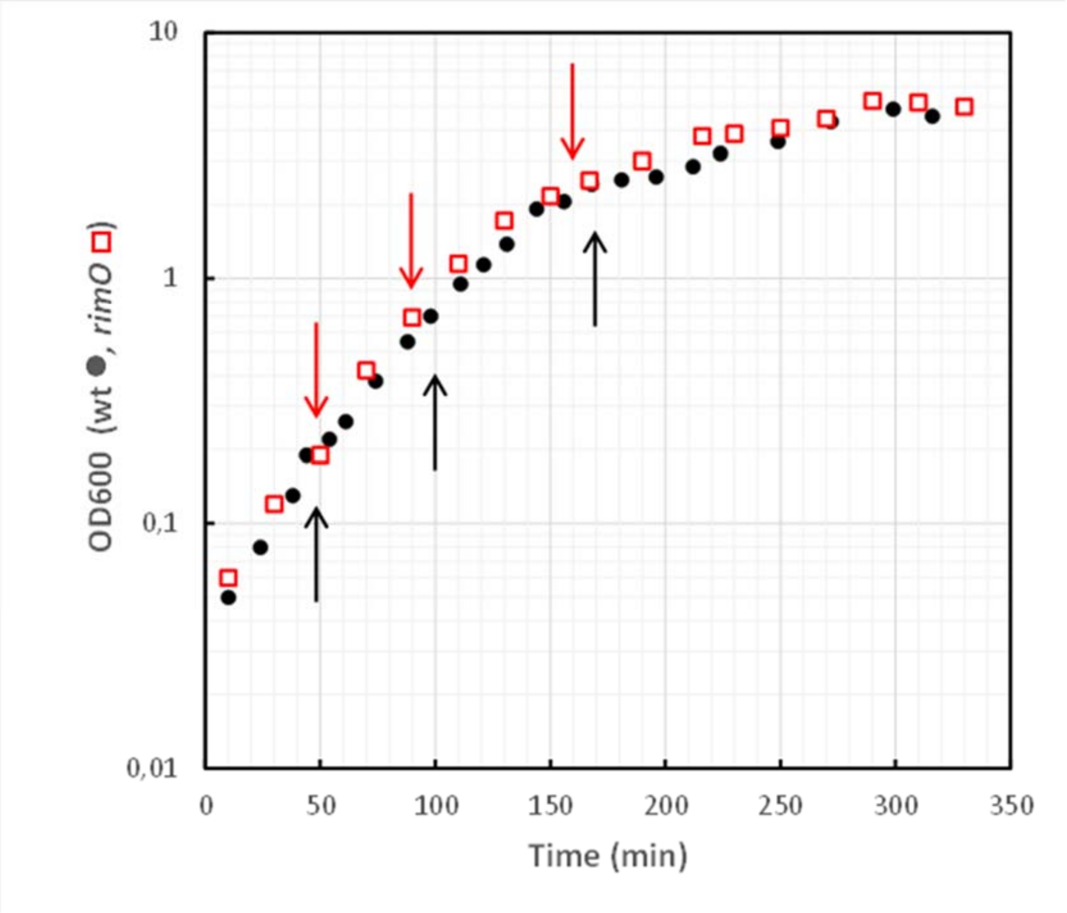

Figure S1. Bacterial strains TC5540 (wt) and TC5541 (*rimO*) were grown in LB medium at 37 ºC. Samples were taken for MS analysis at the points indicated by arrows, at OD600 of 0.2, 0.7 and 2.5 and processed as described in the experimental section.

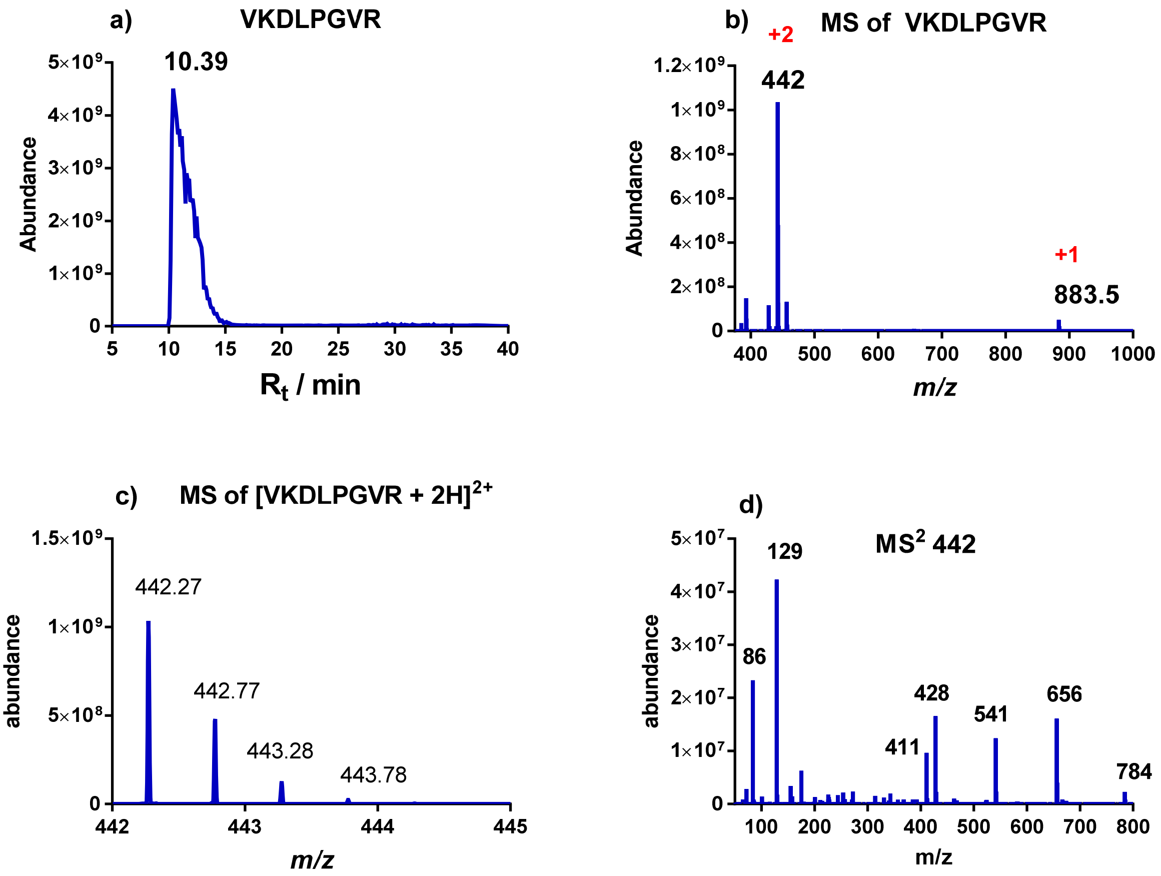

Figure S2: Commercially purchased non-thiomethylated VKDLPGVR used as a standard reference. a) HPLC chromatogram a sample of VKDLPGVR with observed retention time of the peptide peak at 10.39 min. b) ESI-MS spectra of VKDLPGVR showing the single and double charged ions of the peptide . c) Illustrates the ^{13}C isotope distribution (mass difference of 1/2) that proves double charge ion (+2) state. d) Chromatogram shows the MS^2 of *m/z* =442. The following fragment ions are observed: 129 (iminium ion of R), 428 (y4), 541 (y5), 656 (y6).

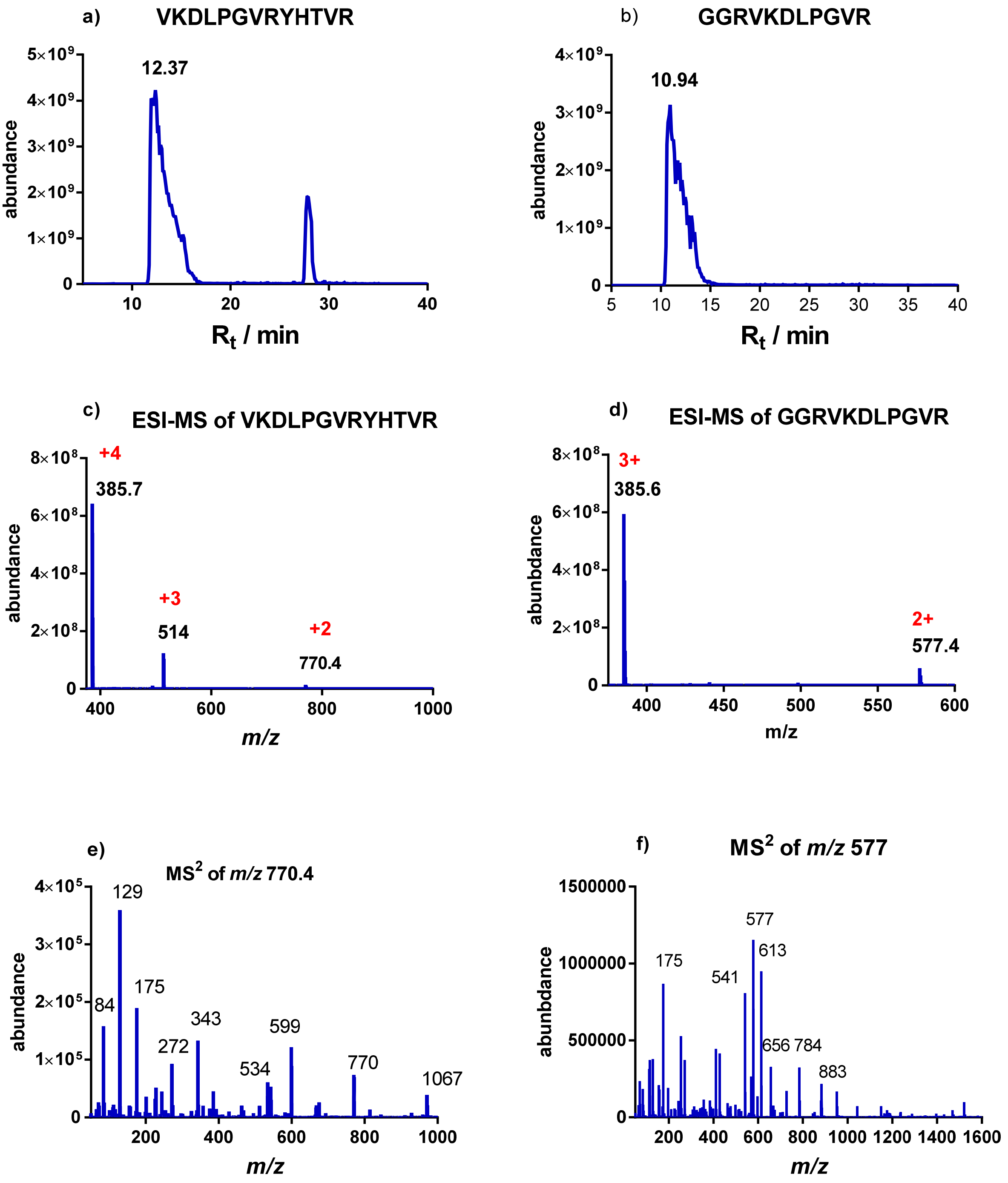

Figure S3: Commercially purchased non-thiomethylated two miscleaved S12 peptides containing D88. a), b) HPLC chromatograms of the two peptides VKDLPGVRYHTVR and GGRVKDLPGVR c),d) ESI-MS spectra of the two peptides. e), f) MS^2 of the double charged ions $[M+2H]^{2+}$ of the two reference peptides.

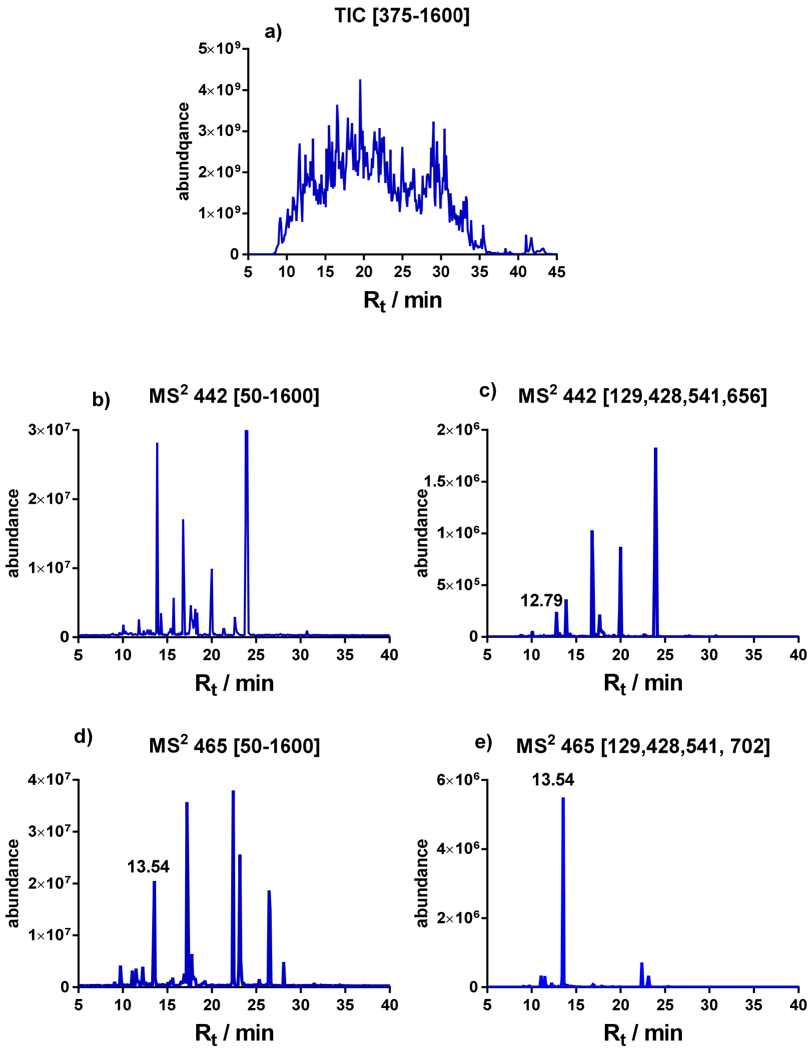

Figure S4 Targeted LC-MS analysis of a trypsin treated wild type bacterial *E. coli* sample. a) Total Ion Chromatogram (TIC) in the *m/z* interval [375-1600]. b),c) Chromatograms of the non thiomethylated VK**D**LPGVR peptide sample with the MS^2 filters, MS^2 442.27@hcd 35 [50-1600] and MS^2 442.27@hcd 35 [129,428,541,656], respectively. d), e) Chromatograms obtained of the thiomethylated VK**D**LPGVR peptide with the MS^2 filters, MS^2 442.27@hcd 35 [50-1600]MS^2 465.26@hcd 35 [129, 428, 541,702], respectively.

Table S1: Percent S12 thiomethylation in wild type (wt), *rimO* and ygfZ bacterial strains grown at 30-42 ºC and harvested at different bacterial growth phases.

Bacterial strain	Geno-type	OD_{600}	t/ºC	Biological Replicates	Area VK**D**LPGVR	Area VK**D**LPGVR	***VKDLPGVR*** / ***VKDLPGVR***
TC5540	wt	0.2	37	A	9.63E06	8.52E05	11.3
				B	3.45E07	1.48E06	23.3
				C	2.68E07	1.07E06	24.9
"	"	0.7	37	A	3.36E07	1.30E06	25.9
				B	2.59E07	5.05E05	51.0
				C	1.76E07	1.08E06	16.3
"	"	2.5	37	A	1.50E07	2.54E05	59.0
				B	1.21E07	0	-
				C	2.00E07	2.25E05	88.7
TC5542	RimO	0.2	37	A	0	1.76E07	0.0
				B	0	2.14E07	0.0
				C	0	1.62E07	0.0
"	"	0.7	37	A	0	2.07E07	0.0
				B	0	1.51E07	0.0
"	"	2.5	37	A	0	4.81E06	0.0
				B	0	3.86E06	0.0
				C	0	3.24E06	0.0
TC5540	wt	0.2	30	A	1.96E07	8.63E05	22.7
				B	3.98E07	2.14E06	18.6
				C	2.91E07	4.57E05	63.8
"	"	0.2	42	A	1.66E07	1.36E06	12.3
				B	1.77E07	3.98E05	44.4
				C	2.91E07	4.57E05	63.8
TC5541	ygfZ	0.2	30	A	2.16E05	1.17E07	0.0
				B	1.40E05	1.71E07	0.0
				C	0	7.51E06	0.1
				D	0	1.26E07	0.0
				E	0	1.34E07	0.0
				G	4.37E05	7.23E06	0.1
TC5541	ygfZ	0.2	42	A	7.86E05	1.55E07	0.1
				B	6.58E05	1.50E07	0.0
				C	9.04E05	1.40E07	0.1
				D	0	1.47E07	0.0
				E	1.49E04	1.37E07	0.0
				G	0	9.47E06	0.0
